# Thrombotic microangiopathy associated with lenvatinib therapy

**DOI:** 10.1530/EO-22-0078

**Published:** 2023-02-14

**Authors:** Macarena Contreras Angulo, Belén García Izquierdo, Laura Armengod Grao, Nuria Palacios García

**Affiliations:** 1 Department of Endocrinology and Nutrition, Puerta de Hierro University Hospital, Majadahonda, Madrid, Spain

**Keywords:** lenvatinib, thyroid cancer, thrombotic microangiopathy

## Abstract

**Summary:**

Systemic thrombotic microangiopathy (TMA) is a serious condition whose early treatment is essential to reduce morbidity and mortality. TMA with only renal involvement has been associated with tyrosine kinase inhibitors, including lenvatinib, a drug used for certain advanced neoplasms. To date, TMA with systemic involvement associated with this drug has not been described. We present the case of a patient with progressive metastatic thyroid cancer who developed this complication after starting treatment with lenvatinib. We describe the signs and symptoms that led to the diagnosis and the treatment required for her recovery.

**Learning points:**

- Thrombotic microangiopathy (TMA) is a group of disorders characterized by thrombosis in capillaries and arterioles due to an endothelial injury. Both, localized and systemic forms have been described.
- TMA with systemic involvement is characterized by hemolytic anemia, low platelets, and organ damage.
- Vascular endothelial growth factor signaling inhibitors have been associated with TMA, either restricted to the kidney or with systemic involvement.
- Lenvatinib has been rarely associated with TMA. Although only forms with isolated or predominantly renal involvement had been described so far, a predominantly systemic form can occur.
- Lenvatinib-induced systemic TMA must be distinguished from primary forms by measuring ADAMTS-13. Treatment includes discontinuation of the drug and supportive measures.
- When anemia and thrombocytopenia coexist in a patient receiving treatment with lenvatinib, a peripheral blood smear to exclude TMA is recommended

## Background

Lenvatinib is a multikinase inhibitor used in the treatment of locally advanced or metastatic differentiated thyroid carcinoma when progression is evident and other treatment alternatives are not viable (Schlumberger *et al.* 2015, Haugen *et al.* 2016).

The adverse effects more often associated with lenvatinib are asthenia, hyporexia, weight loss, increased blood pressure, diarrhea, and hand-foot syndrome. Analytical alterations such as hypocalcemia or hypomagnesemia, proteinuria, and thrombocytopenia are also frequent (European Medicine Agency 2022). More serious adverse effects occur with low frequency according to the information provided by clinical trials. However, as the population exposed to lenvatinib increases, rare but serious adverse events not identified in these studies may become apparent.

Thrombotic microangiopathy (TMA) is a serious and potentially life-threatening complication. Primary and secondary forms have been described, the latter often associated with exposure to certain drugs. Drug-induced TMA is estimated to account for 10–13% of all causes of TMA and 20–30% of all secondary forms, although this proportion seems to be underestimated.

There are two types of drug-induced TMA: type I is mediated by an immune mechanism and is associated with drugs such as oxaliplatin or interferon; type II is related to a cumulative effect that produces a progressive blockade of different pathways involved in the maintenance of endothelial homeostasis. (Mazzierli *et al.* 2023). In particular, anti-vascular endothelial growth factor (VEGF) drugs play a fundamental role in renal endothelial–podocyte complex physiology (Mazzierli *et al.* 2023). Dysregulation of this complex gives rise to a particular clinical entity described as ‘renal-limited TMA’. (Mazzierli *et al.* 2023). Multityrosine kinase inhibitors (TKIs), including lenvatinib, are anti-VEGF agents and have recently been recognized as type II TMA-causing drugs with a definite causal association (Mazzierli *et al.* 2023). Accordingly, most of the lenvatinib-induced TMA cases described in the literature show exclusively renal involvement. To our knowledge, there is no evidence of lenvatinib-induced TMA with predominant systemic involvement.

We present the case of a patient who had lenvatinib-induced TMA with noticeable systemic involvement and only mild kidney damage.

## Case presentation

A 72-year-old woman, with a history of idiopathic thrombopenic purpura (ITP) in youth, arterial hypertension, and type 2 diabetes mellitus, was diagnosed with a papillary thyroid carcinoma pT3N1. She was treated by surgery (total thyroidectomy plus central lymphadenectomy) and radioiodine, after which she achieved an incomplete biochemical response due to an increasing concentration of anti-thyroglobulin antibodies. Three years after diagnosis, cervical lymph node metastases and bilateral pulmonary metastases were discovered. Simultaneously the patient was diagnosed with a right renal carcinoma for which she underwent a right nephrectomy. One month after the nephrectomy, she received treatment with 5550 MBq (150 mCi) of I^131^, the post-treatment scan showing no uptake in the known metastatic lesions. Given this finding and the history of renal neoplasia, a biopsy of a pulmonary lesion was performed which confirmed the thyroid as the origin of the lung metastases. In the following months, disease progression was observed, leading to the initiation of systemic treatment with lenvatinib at a dose of 24 mg/day. Two weeks later, the patient had experienced an increase in blood pressure as the only adverse effect, and hematological and biochemical parameters were normal.

One month after starting lenvatinib, the patient went to the emergency room complaining of paresthesia in the extremities, asthenia, abdominal pain, nausea, and increasing dyspnea at rest for the previous 3 days.

## Investigation

The physical examination was unremarkable and the laboratory tests showed significant anemia (hemoglobin 8.9 g/dL (NV: 12–16), MCV 79 fL (82–97)) with a high percentage of reticulocytes (5.99% (0.5–2.5)), severe thrombocytopenia (18,000 platelets/μL (150,000–400,000)), severe hypocalcemia (5.6 mg/dL ((8.7–10.3), and hypomagnesemia (0.20 mg/dL (0.6–1.1)). Renal function was mildly impaired at baseline, as shown by a creatinine level of 1.18 mg/dL (0.5–0.9) and a glomerular filtration rate (estimated by a Chronic Kidney Disease Epidemiology Collaboration method (CDK-EPI)) of 46 mL/min/1.73 m^2^. Initial Lactate dehydrogenase (LDH) levels were 889 U/L (120–246) and free haptoglobin levels were 58 mg/dL (75–225). A peripheral blood smear showed the presence of 4.1% schistocytes, leading to the diagnosis of TMA. Isolated red blood cells were found in urinalysis. Urine protein was not measured at admission.

## Treatment

**Figure 1:**
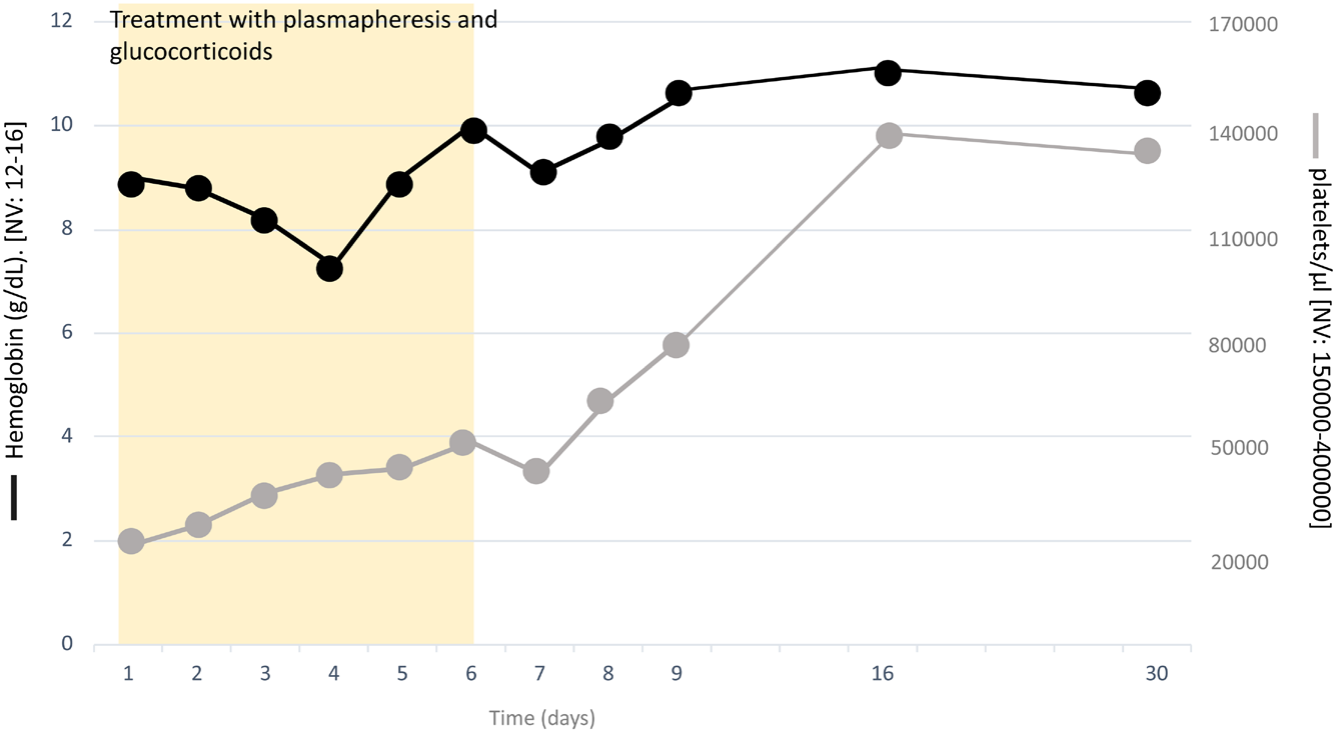
Evolution of hemoglobin and platelet values from diagnosis to recovery.

Prompt treatment with plasmapheresis and prednisone at a dose of 1 mg/kg body weight was started, intravenous calcium and magnesium were infused, lenvatinib was stopped, and a blood sample was taken to measure the activity of ADAMTS-13, an enzyme related to TMA pathogenesis. The patient's symptomatology progressively subsided during the first hours of treatment. In the following days, the patient underwent daily plasmapheresis sessions and continued to receive glucocorticoids. Hemoglobin and platelet levels progressively increased from the beginning of treatment (Figure 1). Urine protein concentration, determined on the fifth day of admission, was slightly elevated (0.22 g/24 h (NV: 0.05–0.12). Complement was not assessed and no renal biopsy was performed.

## Outcome and follow-up

The quantification of ADAMTS-13 activity was available on the sixth day of admission and showed a value of 67.7% (NV: 40–124%). In view of this result, glucocorticoids and plasmapheresis sessions were discontinued, and the patient was discharged on the eighth day of admission with the diagnosis of TMA secondary to lenvatinib. At discharge, she had a hemoglobin level of 11 g/dL and a platelet count of 76,000/μL, which rose to 137,000/μL in the following week.

## Discussion

The name TMA refers to a group of entities characterized by a disruption of the vascular endothelium leading to thrombus formation in the microcirculation. Platelet consumption/trapping in thrombi and destruction of red blood cells by turbulence originating in the microcirculation result, respectively, in thrombocytopenia and hemolytic anemia with schistocytes in peripheral blood. Ischemia secondary to capillary occlusion is responsible for the dysfunction of many organs, mainly the kidney and the nervous system, which often accompanies this disorder. In the absence of therapy, TMA mortality is high, so early treatment is essential.

TMA can be primary or secondary entities. The primary forms (whether familial or acquired) are due to the presence of antibodies against ADAMTS-13 metalloprotease, which cause a decrease in the activity of this enzyme (Moake 2002, Contreras *et al*. 2015). The function of ADAMTS-13 is to break down the von Willebrand factor (vWF) polymers synthesized by endothelial cells into monomers. In absence of this enzyme, the vWF polymers do not cleave and remain anchored to the endothelial cell membrane, inducing local thrombus formation due to their high affinity for platelets. The complement system is one of the final effectors of endothelial damage, and mutations in several complement genes are clearly associated with primary TMA, suggesting an underlying genetic predisposition. Treatment consists of glucocorticoids to suppress the production of anti-ADAMTS13 antibodies, and plasmapheresis to remove circulating antibodies and provide the deficient enzyme.

Secondary forms are due mainly to autoimmune diseases, neoplasms, infections, and drugs. The pathogenesis of secondary forms is heterogeneous, and the role of ADAMTS-13 and complement activation in this setting is unclear. In particular, drug-induced TMA can be classified into two subgroups according to the pathogenic mechanism: immune-mediated (idiosyncratic) and dose-related/toxic (Mazzierli *et al.* 2023). In immune-mediated TMA (also known as type I), antibodies against ADAMTS-13 may be involved in a few cases although antiplatelet antibodies have been described in most reports. Typically, systemic symptoms occur within 21 days of starting the drug. In dose-related TMA (also known as type II), the most frequent form of drug-induced TMA, cumulative toxicity alters the maintenance of endothelial homeostasis through direct endothelial damage, interference with signal protein and/or its receptor (such as the VEGF pathway), interference with signal transducers (such as the mTOR pathway), or dysregulation of transcription factors. In this form, there is not a clear time onset after drug exposure, classically occurring after months.

Treatment of secondary forms consists of supportive measures and elimination of the etiologic factor, if possible. Due to the limited evidence about their efficacy, plasma exchange and anti-complement therapy do not seem to be indicated in the first line; reserving their use in case of poor response to supportive measures, especially in immune-mediated drug-induced TMA. The clinical history and the evaluation of ADAMTS-13 activity help differentiate between primary and secondary forms, a key fact for the appropriate selection of treatment (Moake 2002, Contreras *et al.* 2015, Mazzierli *et al.* 2023).

The diagnosis of TMA could be made in our case as the presence of severe thrombocytopenia, anemia with reticulocytosis, and schistocytes in the peripheral blood smear are some of the main features of this pathology. Acute renal damage is a prominent finding in this entity (Moake 2002, Contreras *et al.* 2015). Although we lacked a biopsy, our patient presented with a slight deterioration of renal function and increased 24-h urine proteinuria, suggesting that renal damage was present. Given that drug-induced TMA presentation is indistinguishable from other TMA forms, treatment with plasmapheresis and glucocorticoids may be indicated at the beginning in severe systemic forms until the results of ADAMTS-13 activity are obtained, which could take several days. A normal ADAMTS-13 activity allows to rule out primary and immuno-related forms in many cases (Moake 2002, Contreras *et al.* 2015) and to stop plasmapheresis and glucocorticoids, as we did in our case.

One factor implicated in secondary forms of TMA is VEGF. VEGF is produced by the podocytes of the renal glomerulus and is essential for the maintenance and proper function of adjacent endothelial cells. The absence of VEGF leads to endothelial cell death, endothelial barrier disruption, platelet aggregation, and microthrombus formation. VEGF seems to have, therefore, a protective role in the pathogenesis of microangiopathic processes. Cases of TMA with exclusively renal involvement associated with VEGF signaling inhibitors have been described (Eremina *et al.* 2008, Fujita *et al.* 2021, Valério *et al.* 2021, Nakashima *et al.* 2022) Therefore, considering that lenvatinib inhibits, among others, VEGF receptors 1–3, it seems reasonable to attribute to this drug an etiopathogenic role in the development of TMA in our patient. In addition, as recently reported by Nakashima *et al.* (2022), lenvatinib can also cause renal damage by affecting renal tubules through the inhibition of platelet-derived growth factor receptor, another therapeutic target of the drug.

To date, a significant number of TMA cases associated with the development of severe proteinuria and renal damage have been described with the use of different TKIs, including lenvatinib (Hyogo *et al.* 2018, Delsante *et al.* 2021, Fujita *et al.* 2021, Nakashima *et al.* 2022). Most of these cases did not show extrarenal involvement (Hyogo *et al.* 2018, Delsante *et al.* 2021, Fujita *et al.* 2021, Mazzierli *et al.* 2023) and therefore had ‘limited renal TMA’, defined as biopsy-proven renal TMA in the absence of microangiopathic hemolytic anemia and thrombocytopenia. In all the cases described, including ours, the onset of hypertension preceded proteinuria. Delsante *et al.* (2021) reported three cases of localized TMA, in which renal function remained preserved, concluding that close monitoring and early discontinuation of treatment can prevent the deterioration of renal function from progressing. Proteinuria improved at least partially in all three patients, as it did in our case. Hyogo *et al.* (2018) maintained a patient on lenvatinib for almost 1 year despite the appearance of proteinuria, which only improved after treatment discontinuation, thus highlighting the ability of lenvatinib to induce this complication. Renal dysfunction was also present in this patient and improved after the change of therapeutic approach.

In contrast to the cases described earlier, TMA with predominantly systemic involvement was diagnosed in our case. Unlike limited renal TMA, cases of TMA with systemic involvement associated with the use of TKIs are rare, mostly associated with the use of sunitinib and less frequently with imatinib (Valério *et al.* 2021). Regarding lenvatinib, only a patient has been described who developed thrombocytopenia and hemolytic anemia associated with lenvatinib-induced TMA, and even in this case, the renal damage seemed to dominate the clinical picture (Nakashima *et al.* 2022). Thus, no cases with predominantly systemic involvement associated with lenvatinib have been reported. Despite this, several reasons led us to consider lenvatinib as the causative agent in our case: i) normal ADAMST-13 activity, ii) rapid improvement after lenvatinib withdrawal, and iii) biological plausibility given lenvatinib's ability to inhibit VEGF signaling (Moake 2002, Eremina *et al.* 2008, Contreras *et al.* 2015, Hyogo *et al.* 2018, Delsante *et al.* 2021, Valério *et al.* 2021, Nakashima *et al.* 2022). Although advanced neoplasm could be a potential cause of systemic TMA in our case, we consider it unlikely as no cases have been described associated with thyroid cancer in the absence of other recognized etiologic factors.

Whether lenvatinib and other VEGF inhibitors have a pathogenic role on their own or they act as triggers in a previously predisposed subject is not known at present. Also, it is unknown to date why TMA associated with TKIs is accompanied in some cases by systemic or generalized involvement and in other cases by isolated renal involvement. However, in view of the patient's history of ITP, we could speculate that she may have a predisposition to produce antiplatelet antibodies and, therefore, TMA could have been mediated by an immune mechanism rather than a toxic one. The early occurrence and the predominance of systemic manifestations seem to fit with this hypothesis.

On the other hand, the hypomagnesemia and hypocalcemia present in our case are attributable to lenvatinib toxicity and are not directly related to TMA.

Limitations of this case include the absence of renal biopsy to demonstrate the characteristic histological changes of TMA and the delay of the urinalysis, which could not be obtained until the fifth day. In addition, there is no information on the presence of autoimmune activity or other features that could have helped in the differential diagnosis such as complement activation or mutations, both usually negative in lenvatinib-induced TMA (Mazzierli *et al.* 2023).

In conclusion, TMA with predominantly systemic involvement should probably be included in the list of adverse effects of lenvatinib and TKIs in general. This is a serious entity whose early treatment is vital to reduce mortality. Therefore, when anemia and thrombocytopenia coexist in a patient receiving treatment with TKIs, it is advisable, even in the absence of significant renal damage, to examine the peripheral blood smear in order to detect this entity early and take the appropriate measures to minimize its clinical impact.

## Declaration of interest

The authors declare that there is no conflict of interest that could be perceived as prejudicing the impartiality of this case report.

## Funding

This work did not receive any specific grant from any funding agency in the public, commercial or not-for-profit sector.

## Patient consent

Written informed consent for publication of their clinical details was obtained from the patient.

## Author contribution statement

All authors have contributed to the writing of the manuscript and all authors have read and approved the final manuscript.
